# Supplementary material for: Vacuolar Proton-Translocating ATPase May Take Part in the Drug Resistance Phenotype of Glioma Stem Cells
Source: Int J Mol Sci. 2024 Feb 27;25(5):2743. doi: 10.3390/ijms25052743 (PMC10932054; doi:10.3390/ijms25052743)
Supplement: Supplementary file 1 [file ijms-25-02743-s001.zip › ijms-2877010-supplementary.pdf]

Supplementary

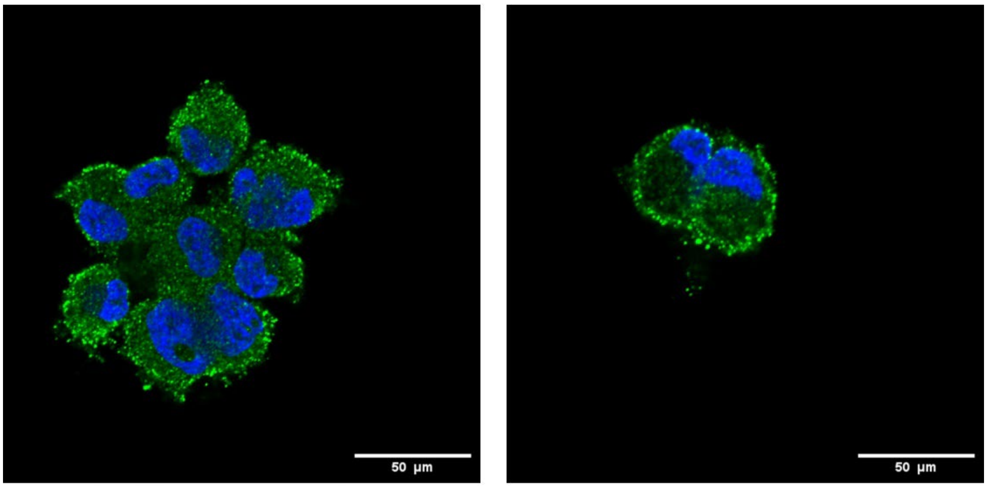

Figure S1. Assessment of V-ATPase localization. The picture shows the immunofluorescence performed on glioma-stem cell (GSC) line 7 as example. V-ATPase can be appreciated in green while nuclei are in blue (DAPI). Images were captured at 40X magnification. Unit of measurement bar = 50 µm.
